# The Diagnosis Performance of the TCM Syndromes of Irritable Bowel Syndrome by Gastroenterologists Based on Modified Simple Criteria Compared to TCM Practitioners: A Prospective, Multicenter Preliminary Study

**DOI:** 10.1155/2020/9507674

**Published:** 2020-07-09

**Authors:** Jun Song, Ling Yang, Shuai Su, Mei-Yu Piao, Bao-Li Li, Lie-Xin Liang, Guo-Wen Zuo, Zhi-Min Tang, Yan-qin Long, Xiao-Li Chen, Ning Dai, Jian-Ling Mo, Yue Yu, Wen-Yong Yu, Mei Zhang, Rong-Quan Wang, Jing Chen, Xiao-Hua Hou

**Affiliations:** ^1^Division of Gastroenterology, Union Hospital of Tongji Medical College, Huazhong University of Science and Technology, 1277 Jiefang Road, Wuhan 430022, China; ^2^Department of Gastroenterology, Tianjin Medical University General Hospital, Tianjin 30052, China; ^3^Department of Traditional Chinese Medicine, Tianjin Medical University General Hospital, Tianjin 30052, China; ^4^Department of Digestion, The People's Hospital of Guangxi Zhuang Autonomous Region, Nanning 530021, China; ^5^Department of Traditional Chinese Medicine, The People's Hospital of Guangxi Zhuang Autonomous Region, Nanning 530021, China; ^6^Department of Gastroenterology, Sir Run Run Shaw Hospital, School of Medicine, Zhejiang University, Hangzhou, Zhejiang 310020, China; ^7^Department of Traditional Chinese Medicine, Sir Run Run Shaw Hospital, School of Medicine, Zhejiang University, Hangzhou, Zhejiang 310020, China; ^8^Department of Gastroenterology, The First Affiliated Hospital of Anhui Medical University, Hefei 230001, China; ^9^Department of Chinese-Western Medicine Integrative Oncology, The First Affiliated Hospital of Anhui Medical University, Hefei 230001, China; ^10^Department of Gastroenterology, The Southwest Hospital of Third Military Medical University, Chongqing 400038, China; ^11^Department of Traditional Chinese Medicine, The Southwest Hospital of Third Military Medical University, Chongqing 400038, China

## Abstract

**Purpose:**

Traditional Chinese medicine (TCM) including Chinese patent medicine has been widely used to treat irritable bowel syndrome (IBS). Syndrome differentiation is the essence of TCM. However, the diagnostic ability of gastroenterologists to detect TCM syndromes in IBS in China remains unknown. The aim of this study was to investigate the ability of gastroenterologists to diagnose the TCM syndromes of IBS based on modified simple criteria compared with TCM practitioners.

**Methods:**

Patients meeting the Rome III criteria for IBS-D or IBS-C were recruited from six tertiary referral centers between January 2016 and December 2017. After learning the diagnosis criteria of the TCM syndromes in IBS, gastroenterologists first diagnosed the syndromes of the enrolled patients. Subsequently, the patients were diagnosed by TCM practitioners. The rate of agreement between the gastroenterologists and TCM practitioners was analyzed. In addition, demographic data and the distribution of TCM syndrome types in IBS were also analyzed.

**Results:**

A total of 178 patients (93 males and 85 females), including 131 patients with IBS-D and 47 patients with IBS-C, were enrolled in this study. The rate of agreement of the syndrome diagnosis between the gastroenterologists and TCM practitioners was 84.3%. The diagnosis consistency rates among IBS-D patients and IBS-C patients were 87.0% and 76.5%, respectively. The most common TCM syndrome type in IBS-D patients was liver depression and spleen deficiency syndrome (27.5%), followed by spleen-yang deficiency syndrome (19.8%). Dryness and heat in intestine syndrome was the most common TCM syndrome in IBS-C patients (57.4%).

**Conclusions:**

Gastroenterologists had good diagnostic agreement with TCM practitioners for diagnosing TCM syndrome types in IBS after learning the diagnostic criteria. This knowledge can aid gastroenterologists in selecting suitable Chinese patent medicine to treat IBS.

## 1. Introduction

Irritable bowel syndrome (IBS), one of the most common functional intestinal diseases, is characterized by recurrent abdominal pain associated with defecation or alterations in stool form and/or frequency that cannot be explained by structural or anatomical abnormalities. IBS is classified into four main subtypes, including IBS with diarrhea (IBS-D), IBS with constipation (IBS-C), IBS with mixed symptoms of diarrhea and constipation (IBS-M), or unsubtyped IBS (IBS-U) according to the patient's predominant disorder in bowel habits [1]. The global prevalence of IBS is approximately 11.2% [2]. IBS substantially influences the health-related quality of life of patients. It is associated with heavy costs to patients and society [3]. The pathophysiology of IBS involves multiple factors, such as brain-gut disorder, gastrointestinal infection, visceral hypersensitivity, increased intestinal permeability, psychiatric and psychological conditions such as anxiety and depression, abnormal gastrointestinal motility, altered gut microbiota, immune dysfunction, and host genetic susceptibility [4, 5].

The main interventions include nonpharmacological and pharmacological treatments [6, 7]. Nonpharmacological interventions include dietary and lifestyle changes such as low FODMAP diet, probiotics, and psychological therapies. The available pharmacological treatments include eluxadoline, rifaximin, alosetron, antispasmodics, bile acid sequestrants, and antidepressants. Because of the complicated pathophysiology mechanisms, the current treatment effect of IBS is suboptimal. A recent US study indicated that neither doctors nor patients were satisfied with the therapeutic effects of IBS treatment [8]. Thus, approximately 50% of patients with IBS seek complementary and alternative medicine, such as traditional Chinese medicine (TCM), moxibustion, and acupuncture, especially in China, Japan, and Korea [9–12].

Numerous clinical studies and meta-analyses have proven that TCM is effective in treating IBS [13–15]. In clinical practice, syndrome differentiation is the essence of TCM. After syndrome differentiation, personalized herbal formulae are prescribed to achieve optimal therapeutic outcomes. However, although various TCMs, including Chinese herbal formulae and Chinese patent medicine, have been used to treat IBS in the clinic, most gastroenterologists are unable to differentiate the TCM syndrome types in IBS. Li et al. found that only 30.5% of studies applied syndrome differentiation, and only 202 studies used syndrome differentiation to prescribe herbal formulae across 735 randomized trials examining the use of Chinese herbal medicine to treat IBS [16]. Gastroenterologists often use the same Chinese patent medicine to treat different TCM syndromes in IBS, which results in poor treatment effects in some patients and worse symptoms for some patients. The main reason is that gastroenterologists do not have theoretical knowledge of TCM and the dialectical classification of IBS. Most gastroenterologists do not understand the complex ancient diagnostic criteria of TCM syndromes in IBS. If the diagnostic criteria of TCM syndromes in IBS are described in modern texts based on the main symptoms, the gastroenterologists may be able to accurately diagnose the TCM syndrome types in IBS.

According to the consensus of experts on the diagnosis and treatment of irritable bowel syndrome in traditional Chinese medicine formulated by the Spleen and Stomach Diseases branch of the Chinese Society of Traditional Chinese Medicine in 2010, there are six main TCM syndromes of IBS: syndrome of spleen deficiency and dampness obstruction, liver depression and spleen deficiency syndrome, spleen-kidney yang deficiency, damp-heat syndrome of spleen and stomach, liver depression qi stagnation, and syndrome of dryness and heat in the intestine [17]. However, this classification did not describe the TCM syndromes of IBS-D and IBS-C. Additionally, some common TCM syndromes of IBS, such as spleen-yang deficiency syndrome, were not included.

Hence, the aim of this study was to investigate the diagnosis of the common TCM syndromes of IBS, including IBS-D and IBS-C, by gastroenterologists. We further assessed the rate of agreement between gastroenterologists and Chinese herbalists for the diagnosis of TCM syndromes in IBS after teaching gastroenterologists the modified simple diagnostic criterion by describing them in modern texts.

## 2. Methods

### 2.1. Trial Design

The study was a prospective, multicenter, double-blinded comparative trial. Ethical approval was obtained from the Clinical Trial Ethics Committee of Tongji Medical College, Huazhong Science and Technology University (2011(025)). Every patient voluntarily provided written informed consent.

### 2.2. Participants

This study was conducted at the outpatient clinic of six tertiary referral centers from January 2016 to December 2017. Consecutive adult patients with IBS were recruited in the study if they were 18–75 years of age. Only those participants meeting the Rome III criteria for IBS-D or IBS-C were recruited [17]. All participants had a normal colonoscopy within the last year.

### 2.3. Diagnostic Criteria of IBS TCM Syndromes Based on Main Symptoms

**Figure 1:**
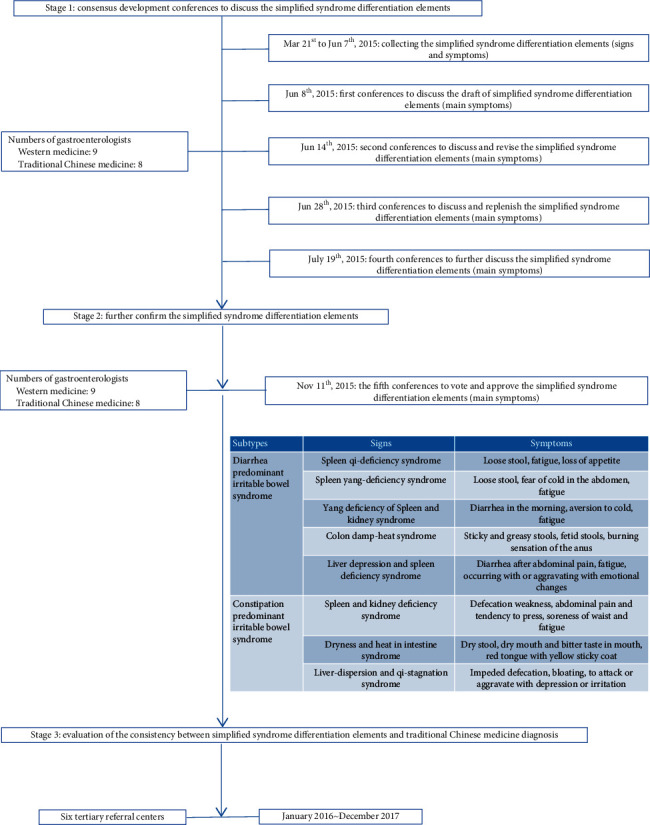
The details of the research procedure.

To enable the gastroenterologists to understand the TCM syndrome diagnostic criteria of IBS, which were described in ancient Chinese medical texts, we first described the TCM syndrome type diagnostic criteria of IBS in modern texts based on the main symptoms. First, two TCM doctors with extensive experience in the field of IBS wrote the first draft of the diagnosis standard of TCM syndrome type of IBS based on main symptoms; this first draft was modified and simple. The criteria were described in modern language and could be understood by gastroenterologists according to the consensus of experts on the diagnosis and treatment of IBS in TCM [18–20]. Seventeen experts, including nine gastroenterologists and eight TCM experts, further discussed and revised the diagnosis standard of IBS TCM syndromes at five different times. The details of the research procedure are shown in Figure 1. Finally, all experts approved the diagnostic criteria of IBS TCM syndromes described in modern medical language. Compared with the TCM syndrome classification of IBS formulated by the Spleen and Stomach Diseases branch of the Chinese Society of Traditional Chinese Medicine in 2010, the expert group further divided the TCM syndrome types of IBS into IBS-D and IBS-C TCM syndrome types according to clinical practice. The details of IBS-D and IBS-C TCM syndrome types are shown in Tables 1 and 2. The main changes in the experts' agreement were that there were more TCM syndrome types of IBS with different subtypes and the updated diagnostic criteria were simpler compared with the previous diagnostic criteria. Compared to the previous TCM syndrome classification, spleen-yang deficiency syndrome and large intestinal damp-heat syndrome are added, and spleen-stomach damp heat is deleted in Table 1. Additionally, deficiency of spleen and kidney syndrome is added in Table 2.

### 2.4. TCM Syndrome Diagnostic Procedure for Patients with IBS

The patients with IBS were divided into IBS-D or IBS-C according to the Rome III criteria. Patients with IBS-M and IBS-U were not enrolled in this study. The TCM syndrome types of the enrolled patients were first diagnosed by gastroenterologists according to the abovementioned criteria. Subsequently, the TCM practitioners diagnosed the TCM syndromes of the patients according to TCM criteria. Gastroenterologists and TCM practitioners were blinded to each other's diagnosis. Each patient visited gastroenterologists and TCM practitioners in the same morning. All the doctors who participated in this study were experienced clinicians who have worked in tertiary referral centers for more than five years.

### 2.5. Study Outcomes

The primary outcome was the agreement between gastroenterologists and Chinese herbalist practitioners, which was defined as the number of the same cases of TCM syndrome type diagnosis divided by the total cases. The secondary outcome was the distribution of TCM syndrome types of IBS-D and IBS-C.

### 2.6. Statistical Analysis

All data are expressed as the mean ± standard deviation (SD) or percentage. SPSS version 17.0 (IBM Corp, Armonk, NY, USA) was utilized for statistical analyses.

## 3. Results

### 3.1. Basic Information of the Participants

A total of 213 patients with IBS, including 104 females and 99 males, were recruited between January 2016 and December 2017. Among the participants, eight patients with IBS-M and 25 patients with IBS-U, according to Rome III criteria, were excluded. Finally, 178 patients, including 131 patients with IBS-D and 47 patients with IBS-C, were analyzed. The basic clinical characteristics of the patients are shown in Table 3.

### 3.2. TCM Diagnostic Agreement Rate between Gastroenterologists and TCM Practitioners

The diagnosis of TCM syndrome in 150 patients with IBS was consistent between gastroenterologists and TCM practitioners. The total agreement rate was 84.3%. A total of 114 patients with IBS-D (87.0%) and 36 patients with IBS-C (76.5%) had the same diagnosis of TCM syndrome. Among the 114 IBS-D patients with the same diagnosis, the number of patients with spleen-qi deficiency syndrome, spleen-yang deficiency syndrome, yang deficiency of spleen and kidney syndrome, large intestinal damp-heat syndrome, and liver depression and spleen deficiency syndrome was 17, 25, 18, 24, and 30, respectively. Among the 36 IBS-C patients with the same diagnosis, the number of patients with deficiency of spleen and kidney syndrome, dryness and heat in intestine syndrome, and liver depression qi stagnation syndrome was 4, 21, and 11, respectively. The details are shown in Table 4. A total of 17 patients with IBS-D had inconsistent diagnoses between gastroenterologists and TCM practitioners. The most common type of TCM syndrome that was misdiagnosed by gastroenterologists was yang deficiency of spleen and kidney syndrome (nine patients), followed by spleen-qi deficiency syndrome (three patients), liver depression and spleen deficiency syndrome (three patients), large intestinal damp-heat syndrome (one patients), and spleen-yang deficiency syndrome (one case). A total of 11 patients with IBS-C had inconsistent diagnoses. The most common types of TCM syndrome misdiagnosed by gastroenterologists were dryness and heat in the intestine syndrome (six patients), deficiency of the spleen and kidney syndrome (4 patients), and liver depression qi stagnation syndrome (1 patients).

### 3.3. Constitution of TCM Syndromes in Patients with IBS

**Figure 2:**
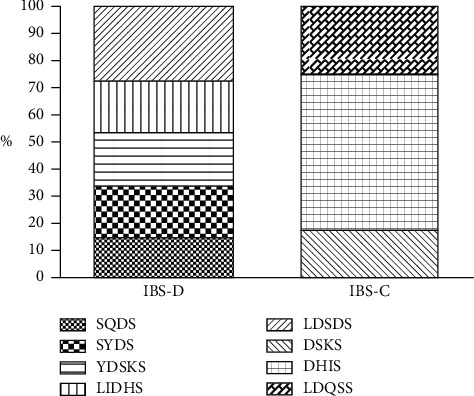
TCM syndrome constitution of IBS- D and IBS-C. SQDS: spleen-qi deficiency syndrome; SYDS: spleen-yang deficiency syndrome; YDSKS: yang deficiency of spleen and kidney syndrome; LIDHS: large intestinal damp-heat syndrome; LDSDS: liver depression and spleen deficiency syndrome; DSKS: deficiency of spleen and kidney syndrome; DHIS: dryness and heat in intestine syndrome; LDQSS: liver depression qi stagnation syndrome.

There were five main TCM syndromes in the patients with IBS-D and three main TCM syndromes in patients with IBS-C. The most common TCM syndrome in IBS-D patients was liver depression and spleen deficiency syndrome (27.5%), followed by spleen-yang deficiency syndrome (19.8%). Dryness and heat in intestine syndrome was the most common TCM syndrome in patients with IBS-C. The different constitutions of TCM syndrome in IBS-D and IBS-C are shown in Figure 2.

## 4. Discussion

To the best of our knowledge, this is the first study to investigate the performance of gastroenterologists in diagnosing TCM syndrome in patients with IBS after systematically learning the TCM diagnostic criteria of IBS based on the main symptoms. There was good agreement between the gastroenterologists and TCM herbalists for the TCM syndrome diagnosis in IBS, especially for patients with IBS-D.

IBS belongs to the categories of diarrhea, abdominal pain, and constipation in TCM. According to TCM, the main causes of IBS are emotional disorders, irregular diets, and external pathogenic factors. The main pathogenesis is weakness of the spleen and stomach, loss of the liver, stagnation of the liver, and deficiency of the spleen [20]. Chinese herbal medicine (CHM) including Chinese patent medicine has been widely used to treat IBS. The effects of TCM on IBS have been confirmed by some systematic reviews and meta-analyses [21, 22]. A recent meta-analysis indicated that TCM showed significant improvement in overall clinical efficacy, compared with cisapride and mosapride, and alleviated abdominal pain, defecation frequency, and stool form compared to the control group. TCM showed greater clinical efficacy in the treatment of IBS-C than cisapride and mosapride, which indicated that TCM was superior to gastrointestinal motility drugs for the treatment of IBS-C [23]. Bensoussan et al. performed a prospective, controlled study and found that CHM improved symptoms of IBS-C, increased bowel satisfaction, and stool consistency and reduced straining and hard lumpy stools, compared with placebo [24]. A meta-analysis performed by Zhu et al. indicated that CHM improved global symptoms, abdominal pain, diarrhea, and pain thresholds compared with placebo and concluded that CHM was an effective and safe treatment for IBS-D [25].

Syndrome differentiation is the core principle of TCM in treating diseases including IBS. Different syndromes of IBS should be treated with different CHMs to achieve the best therapeutic effects [26]. Indeed, numerous studies found that CHMs had obviously different effects on different TCM syndromes in IBS. TCM treatment based on syndrome differentiation was more effective in IBS [27]. However, few doctors prescribed CHM to treat IBS based on TCM syndrome differentiation [16]. For example, gastroenterologists always used the same Chinese patent medicine to treat all IBS types. In fact, the application of Chinese patent medicine is usually only for IBS patients with particular syndromes. Therefore, exact syndrome diagnosis is very important when using CHM to treat IBS. The diagnosis of TCM syndromes is based on four diagnostic methods, namely, inspection, smelling, questioning, and pulse taking. The classification of TCM syndromes is based on clinical manifestations, including symptoms and TCM signs. However, the diagnostic criteria of TCM syndromes are poorly defined and difficult to understand by gastroenterologists [28]. Therefore, we organized TCM and Western medicine experts in the field of IBS to discuss and formulate TCM syndrome type diagnostic criteria based on the main symptoms. These criteria were described with modern language, so that gastroenterologists could understand the contents and perform syndrome differentiation in clinical practice. According to the consensus developed by the Spleen and Stomach Subcommittee of the China Association of Traditional Chinese Medicine, the common TCM syndromes of IBS-D and IBS-C were described with modern language (Tables 1 and 2) based on the main symptoms. Using these criteria, this study found that the diagnostic agreement rate between gastroenterologists and TCM practitioners was 84.3%, which indicated that most gastroenterologists could correctly diagnose the TCM syndromes in IBS after learning the criteria. The agreement rate was 87.0% among patients with IBS-D and 76.5% among patients with IBS-C, which indicated that gastroenterologists were more likely to master the diagnostic criteria of TCM syndromes for IBS-D.

This study also found that the most misdiagnosed syndrome was yang deficiency of the spleen and kidney among the IBS-D TCM syndromes, and dryness and heat in intestine among the IBS-C TCM syndromes. In the 17 inconsistent cases of IBS-D, 9 patients were diagnosed with yang deficiency syndrome of the spleen and kidney by TCM practitioners; however, 5 patients were diagnosed with spleen-qi deficiency syndrome, 2 patients were diagnosed with liver depression and spleen deficiency syndrome, and 2 patients had undetermined symptoms according to the gastroenterologists. The results indicated that it is very difficult for gastroenterologists to distinguish spleen and kidney yang deficiency from spleen-yang deficiency. Indeed, diagnostic criteria of spleen and kidney yang deficiency and spleen-yang deficiency are very similar. The spleen and kidney yang deficiency included the clinical characteristics of deficiency of spleen yang and deficiency of kidney yang. Diarrhea of the spleen and kidney yang deficiency often occurs in the early morning. In addition, 3 cases of spleen-qi deficiency syndrome were misdiagnosed as deficiency of spleen yang (2 patients) and liver depression and spleen deficiency syndrome (1 patient) by gastroenterologists. The main difference was that spleen-yang deficiency syndrome included fear of cold in the abdomen. Three cases of liver depression and spleen deficiency syndrome were misdiagnosed as syndrome of yang deficiency of spleen and kidney (2 patients) and spleen-qi deficiency syndrome (1 patient) by the gastroenterologists. The main difference was that liver depression and spleen deficiency syndrome often occurred or was aggravated by emotions changes. In the 11 inconsistent cases with IBS-C, 6 patients were diagnosed as syndrome of dryness and heat in the intestine by TCM practitioners, which was misdiagnosed as syndromes of yang deficiency of spleen and kidney (5 patients) and liver depression qi stagnation (1 patient) by gastroenterologists. The main diagnostic criteria of syndrome of dryness and heat in the intestine of IBS-C included dry stool, dry mouth, bitter taste in the mouth, and red tongue with a yellow sticky coat. Four cases were diagnosed as syndrome of yang deficiency of the spleen and kidney by TCM practitioners; however, 2 patients were misdiagnosed with liver depression qi stagnation, and 2 patients had undetermined syndrome type according to the gastroenterologists. The results indicated that the gastroenterologists did not master the diagnostic skills of TCM, such as how to observe the tongue coating, or symptoms other than constipation related symptoms. Some gastroenterologists also lack the ability to diagnose the syndrome types after integrating all of the symptoms. Gastroenterologists should further learn and master the key points of these syndrome diagnostic criteria.

Finally, 178 patients were analyzed in this study. There were slightly more males than females in this study (93 males, 85 females). The global prevalence of IBS in women is significantly higher than that in men; [29, 30] however, most studies found no obvious difference in prevalence between males and females [31]. This study also showed similar results. Another interesting finding of this multicenter research was the syndrome distribution of IBS. This study indicated that liver depression and spleen deficiency syndrome and spleen-yang deficiency syndrome were the most common ones. Dryness and heat in intestinal syndrome was the most common in IBS-C. A review listed more than 40 TCM syndromes of IBS that were identified from different studies. The most common syndromes of IBS in the review were liver depression and spleen deficiency, spleen-stomach weakness, and spleen-kidney yang deficiency [16]. Our results were similar to those of the review.

This study had some limitations. First, the TCM syndrome classification and diagnostic standards of IBS in this study were formed through expert discussion. The expert agreement on TCM syndrome classification lacked strict research procedures, such as the Delphi method and comprehensive evaluation. Otherwise, there was no preliminary validation test for the simplified syndrome differentiation elements. Therefore, the classification was not the final version. Second, although this study was performed in six tertiary referral centers, the effective sample size was only 178 patients. The number of enrolled patients in this study is insufficient to study the TCM syndrome classification criteria in IBS. Moreover, only IBS-D or IBS-C patients were included. Therefore, the results of the study should be interpreted cautiously. Third, not all IBS TCM syndromes were included in this study, which may have resulted in misdiagnosis when the gastroenterologists performed syndrome differentiation.

In summary, this study preliminarily confirmed that gastroenterologists can diagnose common TCM syndromes of IBS, especially IBS-D, after learning the criteria of TCM syndromes based on the main symptoms. Additional multicenter and large-scale studies are needed to confirm these findings.

## Figures and Tables

**Table 1:** IBS-D TCM syndrome type diagnostic criteria.

	Corresponding syndromes	Diagnostic criteria
Deficiency syndrome	Spleen-qi deficiency syndrome	Loose stool
		Fatigue
		Loss of appetite
	Spleen-yang deficiency syndrome	Loose stool
		Fear of cold in the abdomen
		Fatigue
	Yang deficiency of spleen and kidney syndrome	Diarrhea in the morning
		Aversion to cold
		Fatigue
Excess syndrome	Large intestinal damp-heat syndrome	Sticky stool
		Foul and smelly stool
		Burning of the anus
Syndrome of intermingled deficiency and excess	Liver depression and spleen deficiency syndrome	Diarrhea with abdominal pain
		Fatigue
		Occurring with or aggravating with emotional changes

**Table 2:** IBS-C TCM syndrome type diagnostic criteria.

	Corresponding syndromes	Diagnostic criteria
Deficiency syndrome	Deficiency of spleen and kidney syndrome	Defecation weakness
		Abdominal pain and tendency to press
		Soreness of waist and fatigue

Excess syndrome	Dryness and heat in intestine syndrome	Dry stool
		Dry mouth and bitter taste in mouth
		Red tongue with yellow sticky coat
	Liver depression qi stagnation syndrome	Impeded bowel movement
		Abdominal distension
		Occurring with or aggravated by emotions changes

**Table 3:** Basic information of the participants.

	IBS-D	IBS-C
Patients, n	131	47

Man/female, n	70/61	23/24

Age, mean (SD), years	41.3 ± 8.6	39.6 ± 8.2

BMI, kg/m^2^ (SD)	23.8 ± 2.4	23.4 ± 2.7

Occupation, *n* (%)		
Worker	39 (29.7)	14 (29.8)
Farmer	1 (0.8)	2 (4.3)
Office worker	48 (36.6)	13 (27.6)
Civil servant	6 (4.6)	5 (10.6)
Freelance worker	8 (6.1)	2 (4.3)
Medical worker	4 (3.1)	1 (2.1)
Self-employed businessman	25 (19.1)	10 (21.3)

Smoking, *n* (%)		
No	81 (61.8)	32 (68.1)
Occasionally	18 (13.8)	8(17)
Frequently	32 (24.4)	7 (14.9)

Drinking, *n* (%)		
No	77 (58.8)	29 (61.7)
Occasionally	25 (19.1)	12 (23.4)
Frequently	29 (22.1)	7 (14.9)

**Table 4:** Agreement rate between gastroenterologists and TCM doctors in TCM syndrome diagnosis.

	Consistent cases (*n*)	Inconsistent cases (*n*)	Agreement rate (%)
IBS-D	114	17	87.0
IBS-C	36	11	76.5
Total	150	29	84.3

## Data Availability

The data used to support the findings of this study are available from the corresponding author upon request.
